# Bradykinin Release Avoids High Molecular Weight Kininogen Endocytosis

**DOI:** 10.1371/journal.pone.0121721

**Published:** 2015-03-30

**Authors:** Igor Z. Damasceno, Katia R. B. Melo, Fabio D. Nascimento, Daianne S. P. Souza, Mariana S. Araujo, Sinval E. G. Souza, Misako U. Sampaio, Helena B. Nader, Ivarne L. S. Tersariol, Guacyara Motta

**Affiliations:** 1 Departamento de Bioquímica, Escola Paulista de Medicina, Universidade Federal de São Paulo (UNIFESP), São Paulo, SP, Brasil; 2 Programas de Biomateriais e Biotecnologia, Universidade Anhanguera de São Paulo (UNIAN SP), São Paulo, SP, Brasil; 3 Departamento de Biofísica, Escola Paulista de Medicina, Universidade Federal de São Paulo (UNIFESP), São Paulo, SP, Brasil; Emory University, UNITED STATES

## Abstract

Human H-kininogen (120 kDa) plays a role in many pathophysiological processes and interacts with the cell surface through protein receptors and proteoglycans, which mediate H-kininogen endocytosis. In the present work we demonstrate that H-kininogen containing bradykinin domain is internalized and different endogenous kininogenases are present in CHO-K1 cells. We used CHO-K1 (wild type) and CHO-745 (mutant deficient in proteoglycans biosynthesis) cell lines. H-kininogen endocytosis was studied using confocal microscopy, and its hydrolysis by cell lysate fraction was determined by immunoblotting. Bradykinin release was also measured by radioimmunoassay. H-kininogen interaction with the cell surface of CHO-745 cells resulted in bradykinin release by serine proteases. In CHO-K1 cells, which produce heparan and chondroitin sulfate proteoglycans, internalization of H-kininogen through its bradykinin domain can occur on lipid raft domains/caveolae. Nevertheless bradykinin-free H-kininogen was not internalized by CHO-K1 cells. The H-kininogen present in acidic endosomal vesicles in CHO-K1 was approximately 10-fold higher than the levels in CHO-745. CHO-K1 lysate fractions were assayed at pH 5.5 and intact H-kininogen was totally hydrolyzed into a 62 kDa fragment. By contrast, at an assay pH 7.4, the remained fragments were 115 kDa, 83 kDa, 62 kDa and 48 kDa in size. The antipain-Sepharose chromatography separated endogenous kininogenases from CHO-K1 lysate fraction. No difference was detected in the assays at pH 5.5 or 7.4, but the proteins in the fraction bound to the resin released bradykinin from H-kininogen. However, the proteins in the unbound fraction cleaved intact H-kininogen at other sites but did not release bradykinin. H-kininogen can interact with extravascular cells, and is internalized dependent on its bradykinin domain and cell surface proteoglycans. After internalization, H-kininogen is proteolytically processed by intracellular kininogenases. The present data also demonstrates that serine or cysteine proteases in lipid raft domains/caveolae on the CHO cell can hydrolyze H-kininogen, thus releasing kinins.

## Introduction

Kinins, including bradykinin, kallidin (Lys-bradykinin) and Met-Lys-bradykinin, are a family of vasoactive and proinflammatory peptides. Kallidin and Met-Lys-bradykinin are converted very rapidly to bradykinin by aminopeptidases [1]. Bradykinin was discovered 65 years ago by Rocha e Silva et al. [2] and possesses multiple biological activities. In addition to being a well-known vasodilator and vasopermeability factor, bradykinin exhibits proangiogenic effects, including the stimulation of cell proliferation, tube formation, and the survival of cultured endothelial cells. Furthermore, bradykinin promotes angiogenesis in the rabbit cornea, the chick embryo chorioallantoic membrane, nude mouse xenograft assays, the rat subcutaneous sponge model, and mouse ischemic hindlimb. Bradykinin is involved in pathological states, including hypertension and inflammation [3].

The precursors of kinins in mammals are high (H) and low (L) molecular weight kininogens, glycoproteins synthesized in liver that exist predominantly in blood plasma but are also found in other body fluids and organs, including the kidney, and in cells such as neutrophils. Kininogens are multifunctional and multidomain glycoproteins related to cystatins (family 1: stefins; family 2: cystatins; family 3: kininogens). Both native forms possess an identical N-terminal heavy chain, which corresponds to domains D1-D2-D3, a short domain D4 (the bradykinin moiety), and because of an alternative splicing of the gene transcript, two different C-terminal light chains; L-kininogen possesses a single D5 domain (D5L), whereas H-kininogen possesses a D5 domain (D5H) as well as a D6 domain. Indeed, both kininogens are encoded by a single gene (also referred to as the K gene) located on chromosome 3 that consists of eleven exons and ten introns [4].

In humans, bradykinin and Lys-bradykinin are generated by the proteolytic cleavage of kininogens by a family of serine proteases called kallikreins produced in plasma and tissue. In the liberation of bradykinin, H-kininogen is a superior substrate of plasma kallikrein, and L-kininogen is a superior substrate of tissue kallikrein. However, both kininogens are substrates to both forms of kallikrein and the liberated bradykinin and Lys-bradykinin molecules are potent vasoactive peptides [5]. Two pharmacologically distinct kinin receptor subtypes have been identified, which are named B1 and B2 receptors, which are members of the G-protein coupled receptor (GPCR) family and are initiators of complex intracellular signaling networks [6].

Human plasma kallikrein and activated factor XII (at a much lower rate) are both capable of the proteolytic digestion of H-kininogen with the subsequent release of bradykinin (proangiogenic) and residual cleaved bradykinin-free H-kininogen (anti-angiogenic) [7], [8]. These proteins exert effects in most tissues of the cardiovascular system throughout the life of an individual. Many actions of these proteins are mediated through the effects of plasma kallikrein, cleaved bradykinin-free H-kininogen and bradykinin to stimulate production of intra- and extracellular messengers and effectors in a variety of cells. There is a large body of evidence demonstrating that the plasma kallikrein-kinin system drives regional blood flow *via* bradykinin-induced B2 receptor activation and promotes intravascular thrombus formation *via* factor XI activation after an injury [9].

Human tissue kallikreins or glandular kallikreins are serine proteases distinguished from plasma kallikrein by their catalytic mechanisms and the proteins targeted for cleavage. Tissue kallikreins are synthesized as inactive pre-proenzymes and are widely expressed in diverse human tissues and cells, including the kidney, pancreas, colon, pituitary gland, erythrocytes, central nervous system, spleen, adrenal glands and neutrophils. Tissue kallikrein cleaves either H-kininogen or L-kininogen at Met-Lys and carboxy-terminus Arg-Ser sites, resulting in the subsequent liberation of the decapeptide Lys-bradykinin. *In vivo*, the decapeptide Lys-bradykinin can be easily converted into the nonapeptide bradykinin by cleavage at the Lys–Arg bond [5].

H-kininogen binds to a wide variety of proteins on the surface of cardiovascular cells in a specific, reversible, and saturable manner and is dependent on Zn^2+^. The cells of the intravascular compartment, including platelets, neutrophils, monocytes, macrophages, astrocytes and vascular smooth muscle cells, assemble the proteins of the plasma kallikrein-kinin system on their surface. Membrane-binding proteins of H-kininogen include the globular head domains of the complement component C1q (gC1qR), urokinase plasminogen activator receptor (u-PAR), and cytokeratin 1(CK1). H-kininogen binding to endothelial cells has been shown to be dependent largely on domain 5 but also on domain 3, and bradykinin links the two sites and contributes to binding in the intact protein [10].

Kininogen binding on the surface of target cells is important for the accumulation of precursor of kinins. When H-kininogen is proteolyzed by plasma kallikrein or other proteases to form its cleaved form without bradykinin, membrane tropomyosin also functions as a binding site uniquely for the bradykinin-free H-kininogen form. Although the proangiogenic activities of the plasma kallikrein-kinin system are mediated by B1R and B2R, a different receptor system(s) may be involved in the inhibition of cell proliferation, adhesion, anti-apoptosis and angiogenesis, and u-PAR has been shown to mediate intracellular signaling [11]. Kolte et al. [12] demonstrated that H-kininogen regulates endothelial cell function in human pulmonary artery endothelial cells in plasma kallikrein-deficient conditions by stimulating a considerable and dose-dependent increase in the intracellular calcium level, which in turn stimulates the production of endothelial nitric oxide and prostacyclin (PGI2).

On the cell surface, H-kininogen binds to heparan sulfate (HS) or chondroitin sulfate (CS) proteoglycans (PGs) [13], [14], and HSPG appear to play a critical role in recruiting kinin precursors from the plasma and in the assembly of components triggering the release of active kinins from their precursors in proximity to their target cells [15]. Indeed, glycosaminoglycans (GAGs) that accumulate in the inflammatory fluids could act as pro- or anti-inflammatory mediators depending on different factors within the cell environment [16]. The H-kininogen interaction with HS on the cell surface results in its endocytosis into acidic vessels [17].

The present study examines the interaction of H-kininogen with cells using tumor epithelial cell lineages Chinese Hamster Ovary, CHO-K1 and CHO-745, which differ from each other with respect to GAG biosynthesis [18]. On the surface of both types of cells, most H-kininogen remains intact, and the endocytosis of bradykinin-containing H-kininogen (120 kDa) but not bradykinin-free H-kininogen is mediated by proteoglycans. In the absence of PG on the cell surface, bradykinin is released mainly by serine proteases. The intact H-kininogen is hydrolyzed by lysate fractions prepared from CHO-K1 cells at pH 5.5 and 7.4, and the lysate fractions purified on antipain-Sepharose demonstrate the presence of different endogenous kininogenases.

## Material and Methods

### Reagents

All chemicals obtained from commercial sources were of the best grade available. SuperSignal West Pico Chemiluminescent Substrate, the biotinylation kit (NHS-LC-Biotin) and EDC (1-etil–3–(3–dimetilaminopropil)carbodiimidacloridrato were purchased from Pierce Biotechnology Inc. (Rockford, IL, USA). The inhibitors captopril, aprotinin, thiorphan, trans-epoxysuccinyl-l-leucylamido-(4-guanidino)butane (E-64), benzamidine, phenylmethylsulfonylfluoride (PMSF), antipain, 1,10-phenanthroline (*o*-phe), goat IgG anti-mouse IgG peroxidase-conjugated and goat IgG anti-rabbit IgG peroxidase-conjugated antibodies were purchased from Sigma-Aldrich Co. (St. Louis, MO, USA). The inhibitor JA-2 [N-[1(R,S)-carboxy-3-phenylpropyl]-Ala-Aib-Tyr-p-aminobenzoate], originally from Ian Smith of the Baker Heart Research Institute, Australia, was kindly provided by Prof. A.C.M. Camargo (Instituto Butantan, São Paulo, SP, Brasil). H-kininogen was obtained from EMD Biosciences Inc. (La Jolla, CA, USA); bradykinin-free H-kininogen was purchased from Enzyme Research Laboratories (South Bend, IN, USA). Lyso Tracker (LT) Red DND-99, 4’-6-diamidino-2-phenylindole dihydrochloride (DAPI), goat IgG anti-mouse FITC-conjugated antibody, and the Alexa Fluor 488 Protein Labeling kit were obtained from Molecular Probes/Invitrogen Detection Technologies (Eugene, OR, USA). Molecular weight standards for SDS-electrophoresis Prestained Broad Range or Kaleidoscope Prestained molecular weight standards were purchased from Bio-Rad Laboratories Ltd. (Hercules, CA, USA). Membrane Immobilon-P transfer membrane (PVDF) was obtained from Millipore Corporation (Billerica, MA, USA). Fluoromont-G was purchased from Electron Microscopy Sciences (Hatfield, PA, USA). FITC-conjugated streptavidin, FITC-conjugated donkey IgG anti-rabbit IgG and Texas Red dye-conjugated streptavidin were obtained from Jackson ImmunoResearch Laboratories, Inc. (West Grove, PA, USA). Fluoromount-G was purchased from Electron Microscopy Sciences (Hatfield, PA, USA). The Densitometer Quick Scan Flur Vis was from Helena Laboratories (Beaumont, KS, USA). Both Sephadex G-25 (PD-10 column) and AH Sepharose 4B resins were obtained from GE Healthcare (Buckinghamshire, UK).

Rabbit IgG anti-caveolin-1-(N-20) was purchased from Santa Cruz Biotechnology, Inc. (Santa Cruz, CA, USA). According to human H-kininogen structure [8] the mouse IgG anti-bradykinin (clone MBK3) was produced against synthetic bradykinin (recognizes the peptide in human H-kininogen R_363_-R_371_) and purchased from Alexis Biochemicals (Lausen, LT, Switzerland); the mouse IgG anti-kininogenD6 (HKL1, against peptide sequence S_543_-M_554_ in H-kininogen domain6) was kindly provided by Prof. Dr. Werner Müller-Esterl (Institute for Biochemistry II, University Hospital, Frankfurt, Germany) [19]; the rabbit IgG anti-human H-kininogen (recognizes whole molecule) was produced following the protocol established by our group [20], and the serum was purified on Immobilized Protein A obtained from Thermo Scientific (Rockford, IL, USA), the rabbit IgG anti-bradykinin produced against synthetic bradykinin recognizes in human H-kininogen bradykinin (R_363_-R_371_), lysyl-bradykinin (K_362_-R_371_) and methionyl-lysyl-bradykinin (M_361_-R_371_) [21]. Tyr-bradykinin and rabbit IgG anti-bradykinin were kindly supplied by Prof. K. Shimamoto (II Department of Internal Medicine, Sapporo Medical University, Sapporo, Japan).

### Cell lines and culture conditions

The epithelial cell lines CHO-K1 and CHO-745 (the mutant deficient in xylosyltransferase) [18] were gifts from Dr. *Jeffrey* D. *Esko* (Department of Cellular and Molecular Medicine, Glycobiology Research & Training Center, University of California-San Diego, La Jolla, California, USA). The protocols used in this study were approved by the Ethics Committee on Research of EPM/UNIFESP (CEP 0016/11). Cells were cultured in a humidified incubator containing 2.5% CO_2_ at 37°C, grown in culture medium [Ham F-12 nutrient mixture medium supplemented with 10% (v/v) heat-inactivated fetal calf serum containing 10 U penicillin and 10 μg/ml streptomycin] and grown to confluence on 60-mm dishes.

### Bradykinin release

A radioimmunoassay was performed to measure bradykinin release according to the procedure described previously [17]. Briefly, CHO-745 cells were grown to confluence on 35-mm dishes, and H-kininogen (200 nM) interaction with cells was analyzed in the presence of two different buffers: (1) HEPES-Tyrode pH 7.35 [135.0 mM NaCl, 2.7 mM KCl, 11.9 mM NaHCO_3_, 0.36 mM NaH_2_PO_4_, 14.7 mM HEPES, 50.0 μM Zn^2+^, 1.0 mM Mg^2+^, 2.0 mM Ca^2+^, 3.5 mg/mL dextrose, 3.5 mg/mL bovine serum albumin (BSA)] or (2) MES-Tyrode pH 5.5 [135.0 mM NaCl, 2.7 mM KCl, 11.9 mM NaHCO_3_, 0.36 mM NaH_2_PO_4_, 14.7 mM MES, 50.0 μM Zn^2+^, 1.0 mM Mg^2+^, 2.0 mM Ca^2+^, 3.5 mg/mL dextrose and 3.5 mg/mL (BSA)]. HK (200 nM) was incubated with cells in the absence (control) or presence of kininase inhibitors (50 nM JA2, 50 nM thiorphan, 2 μM captopril), kininase inhibitors and serine protease inhibitors (10 μM aprotinin, 100 μM benzamidine, 1 mM PMSF) or kininase inhibitors and a cysteine protease inhibitor (9 μM E-64). After 3 h at 37°C, the incubation buffer of each sample was collected and mixed with ethanol (1:20, v/v) for 10 min at 70°C to extract kinin. Solutions were freeze-dried and dissolved in 250 μL egg albumin buffer (0.1% egg albumin in 0.01 M phosphate buffer pH 7.0, 0.14 M NaCl, 0.1% NaN_3_, 30 mM EDTA, 3 mM *o*-phe). Aliquots (50 μL) were incubated with 100 μL of rabbit IgG anti-bradykinin (1:25,000) and [^125^I]-labeled Tyr-bradykinin (100 μL) for 20 h at 4°C. In total, 400 μL of 0.1% bovine γ-globulin in 0.01 M phosphate buffer pH 7.0, 0.14 M NaCl, and 0.1% NaN_3_ and 800 μL of 25% polyethylene glycol 6000 solution were added to the samples, which were incubated for 10 min at 4°C. Finally, the samples were centrifuged at 2,000 *g* for 20 min at 4°C; the supernatants were removed, and the pellets were submitted to radiation counting (Cobra II Auto-Gama, Packard BioScience, CT, USA). The total amount of bradykinin released was calculated, and the data are presented as the mean ± SD, n = 3.

In addition to bradykinin release measured by radioimmunoassay, investigations were conducted to analyze the structure of H-kininogen present in the incubation buffer of CHO-K1 and CHO-745 cells. Briefly, a 35-mm plate containing confluent CHO-K1 or CHO-745 cells was incubated with intact H-kininogen (200 nM) in a single volume of HEPES-Tyrode pH 7.35, without BSA, and in the absence of kininase inhibitors. At the end of a specific time point between 0 min and 3 h, an aliquot of the incubation buffer was removed and mixed with electrophoresis (SDS-PAGE) sample buffer containing the reducing agent β-mercaptoethanol. Following the procedure described below, immunoblotting studies were performed using rabbit IgG anti-H-kininogen (recognizes whole kininogen molecule). After detection by chemiluminescence, the first antigen-antibody complex was removed. The same PVDF membrane was incubated with mouse IgG anti-bradykinin antibody (recognizes R_363_-R_371_ sequence in H-kininogen), and the complex was detected by chemiluminescence. Each figure is representative of the results from three experiments conducted in duplicate.

### Immunocytochemistry and endocytosis of H-kininogen

Immunocytochemistry experiments were performed in fixed cells, and the H-kininogen was biotinylated as reported previously [22]. CHO-K1 or CHO-745 cells were grown on glass coverslips for 3 days at 37°C. The medium was removed, and the cells were washed with serum-free F12 medium added to 50 μM Zn^2 +^. In experiments where caveolin was detected, cells were incubated with biotin-H-kininogen (200 nM) diluted in serum-free F12 medium with 50 μM Zn^2+^ for 1 h at 37°C. The biotin-H-kininogen was removed by aspiration, and the cells were fixed with 2.0% paraformaldehyde in 10 mM sodium phosphate and 150 mM NaCl pH 7.4 (PBS). The cells were subsequently permeabilized with PBS containing 0.01% saponin and 1.0% BSA at room temperature. Rabbit IgG anti-caveolin-1 was added at room temperature, and donkey IgG anti-rabbit IgG-FITC was added at room temperature; the biotin-H-kininogen detection was performed with streptavidin-Texas Red conjugated at room temperature. Cells were incubated with DAPI and mounted on glass slides with Fluoromount-G diluted in PBS (2:1, v/v).

In experiments in which the H-kininogen was detected by mouse IgG anti-bradykinin (recognizes R_363_-R_371_ sequence in H-kininogen), cells were first washed with serum-free F12 medium with 50 μM Zn^2+^ and were incubated with LysoTracker Red DND-99 (500 nM) for 20 min diluted in serum-free F12 medium plus 50 μM Zn^2 +^. Alternatively, cells were pretreated for 24 h with 50 mM sodium chlorate diluted in medium with 50 μM Zn^2+^. After the addition of LysoTracker Red DND-99, H-kininogen (200 nM) was added in the absence or presence of serine protease inhibitors [10 mM aprotinin, 1.0 mM PMSF and 100 mM benzamidine] diluted in serum-free F12 medium plus 50 μM Zn^2+^ for 3 h at 37°C. The H-kininogen was removed by aspiration, and the cells were fixed and permeabilized as described above. The mouse IgG anti-bradykinin (recognizes R_363_-R_371_ sequence in H-kininogen) was added for 1 h, followed by the addition of goat IgG anti-mouse FITC-conjugated antibody at room temperature. The cells were incubated with DAPI and mounted on glass slides with Fluoromount-G. The cells were examined using a scanning confocal microscope.

The endocytosis experiments were performed in real time at 37°C; H-kininogen-Alexa 488 and bradykinin-free H-kininogen-Alexa 488 were prepared as described previously [17]. Briefly, living CHO cells grown on coverslips were washed with serum-free F12 medium plus 50 μM Zn^2+^ and incubated for labeling the endocytic compartments with 0.5 μM LT Red DND-99 for 20 min. After washing with serum-free F12 medium plus 50 μM Zn^2+^, the cells were incubated with H-kininogen-Alexa 488 (80 nM) or bradykinin-free H-kininogen-Alexa 488 (80 nM) diluted in serum-free F12 medium plus 50 μM Zn^2+^. The fluorescent signals of LT Red DND-99 and Alexa 488 (green) as well as the phase contrast micrographs were monitored in real time at 37°C with a confocal laser scanning microscope every 5 min. Each figure is representative of the results from three experiments conducted in duplicate.

The labeled molecules in acidic compartments were analyzed using an inverted confocal laser-scanning microscope (Zeiss LSM-780, Carl Zeiss, Jena, Germany). Donkey IgG anti-rabbit IgG-FITC conjugated and goat IgG anti-mouse FITC-conjugated antibodies were visualized under a 488-nm excitation from a CW Argon ion laser, and its emission was detected at 490–550 nm (green channel). LT Red and streptavidin-Texas Red conjugated antibodies were observed with a HeNe_2_ laser with excitation at 633 nm, and fluorescence emission was detected at 640–670 nm (red channel). Nuclear compartments were stained with DAPI and visualized under 359 nm excitation from a multiphoton laser, and emission was detected at 450–470 nm. Cells were also imaged by differential interference contrast microscopy (DIC) using a HeNe_2_ laser at 633 nm. False color fluorescent images and transmitted light images (1080 × 1080 pixels) were stored, quantified and managed using the resident LSM Image Browser software (Carl Zeiss, Jena, Germany). This allowed fluorescence intensity profiles and colocalization rates to be measured, thereby providing mean (± SD) intensity values for the green and red emission channels of each image.

### Preparation of lysate fractions from CHO-K1 cells

In the preparation of lysate fractions, CHO-K1 cells were grown in 75 cm^2^ flasks to confluence followed by incubation with 3.73 mg/mL pancreatin. The cell suspension was added to the culture medium, the solution was collected in a tube, and the supernatant was discarded after centrifugation. Buffer solution (50.0 mM sodium acetate, pH 5.5 or 20.0 mM HEPES, 5.0 mM NaCl, pH 7.4) was added to the cell pellet, and the number of cells was counted. The cells were washed two more times with the same buffer solution at pH 5.5 or pH 7.4, and chilled buffer (5.0 ml) was added. The cells were ruptured using a vertical sonicator (3 cycles/30 seconds each cycle and 30 seconds stop between each sonication), and the cell lysates were centrifuged at 9,000 *g* for 30 min at 4°C. The lysate fraction was separated and stored at -80°C.

Antipain is an inhibitor that blocks the activity of both serine and cysteine proteases [23]. An antipain-Sepharose resin, prepared according to Toyo-Oka and Masaki [24], was pre-equilibrated with 50 mM sodium acetate, pH 5.5, and the lysate fraction from CHO-K1 cells prepared in the same buffer was mixed with resin for approximately 30 min. Several washings were performed with the same buffer to separate the protein not bound to antipain, and the protein retained to resin was eluted by 50.0 mM sodium acetate, 0.5 M NaCl, pH 5.5. Subsequently, the samples were subjected to gel filtration chromatography on Sephadex G-25 for both fractions retained or not retained by removing the high concentration of salt.

### Kininogenase activity present in the lysate fraction of CHO-K1 cells

The protein concentration was determined by the Bradford method using Coomassie Blue dye G250. In the studies of H-kininogen hydrolysis at different time course, a single volume of sample was prepared by maintaining the proportion of intact H-kininogen (1.0 mg) to CHO-K1 lysate fraction (3.5 mg). All samples were incubated in 50.0 mM sodium acetate, pH 5.5 or 20.0 mM HEPES, 5.0 mM NaCl, pH 7.4 at 37°C. At predetermined terms, an aliquot of incubation sample was withdrawn, and the reactions were stopped with sample buffer for electrophoresis and frozen immediately.

The separation of proteins was performed by sodium dodecyl sulfate polyacrylamide gel electrophoresis (SDS-PAGE) under reducing conditions [25]. After the protein was electroblotted onto a PVDF membrane, a primary antibody and a secondary antibody were used. The antigen-antibody complex was developed with the SuperSignal chemiluminescent substrate [26]. In an attempt to characterize the fragments of H-kininogen, different primary antibodies were used after the first development by stripping the antigen-antibody complex from the PVDF membrane. The membrane was incubated for 30 min at 56°C with a stripping solution [80.0 mM tris pH 7.35 containing 69.0 mM SDS and 0.78% β-mercaptoethanol (v/v)], and the same protocol was repeated by incubation with primary and secondary antibodies and development by chemiluminescence. The intact H-kininogen or fragments were detected with rabbit IgG anti-H-kininogen, mouse IgG anti-bradykinin (recognizes R_363_-R_371_ sequence in H-kininogen) or mouse IgG anti-kininogenD6 (recognizes S_543_-M_554_ sequence in H-kininogen domain6). Each figure is representative of the results from three experiments conducted in duplicate.

### Statistical analysis

The results are shown as mean ± SEM and the statistically significant differences were estimated using analysis of variance and Dunnett’s multiple comparison test accepting a *P*-value of 0.05 or less as statistically significant.

## Results

In this work, we present the results of kinin release in incubation buffer measured by radioimmunoassay after H-kininogen incubation on the surface of CHO-745 cells, which express reduced levels of glycosaminoglycans. The amount of bradykinin in the incubation buffer at pH 7.35 in the presence of kininase inhibitors was two-fold higher than (2.290 ± 0.085 ng/10^6^ cells) the levels in the absence of kininase inhibitors (1.144 ± 0.040 ng/10^6^ cells); in the presence of serine protease inhibitors, bradykinin release was very low (0.079 ± 0.001 ng/10^6^ cells), indicating that bradykinin release was blocked by 97%. However, in these cells, bradykinin release in the presence of cysteine protease inhibitors was not blocked (2.570 ± 0.210 ng/10^6^ cells). Nevertheless, under the same conditions but at pH 5.5, almost no bradykinin release was detected in the incubation buffer of these cells (data not shown).

**Fig 1:**
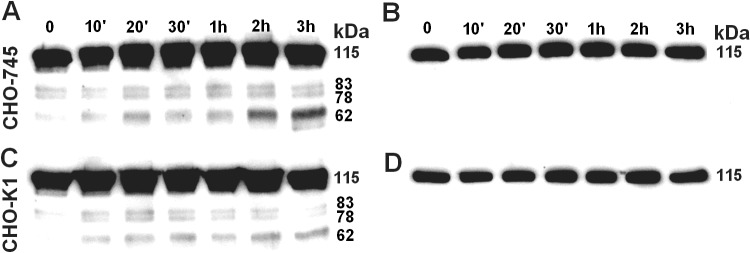
H-kininogen structure determined in the incubation buffer of CHO cells.

The same experiment was performed at pH 7.35, and the structure of H-kininogen in the incubation buffer of CHO-745 and CHO-K1 cells at different time points was verified by immunoblotting; the results are presented in Fig. 1. In Fig. 1A, in CHO-745 cells the rabbit IgG anti-H-kininogen demonstrates partial H-kininogen hydrolysis. In 0 min time point H-kininogen (115 kDa) was detected (85.2 ± 3.9%, n = 5) and a fragment of 62 kDa in size was also detected (4.8 ± 2.5%, n = 5); after 1 h incubation there was partial H-kininogen hydrolysis and the amount of 115 kDa band decreased significantly (70.4 ± 5.9%, n = 5, *P* < 0.05) and the 62 kDa fragment in size increased significantly (17.3 ± 5.3%, n = 5, *P* < 0.05); at the end of incubation (3 h) the H-kininogen 115 kDa in size remained uncleaved but decreased significantly (66.8 ± 8.2%, n = 5, *P* < 0.001) and the 62 kDa fragment in size increased significantly (24.2 ± 7.7%, n = 5, *P* < 0.001). In Fig. 1B, after stripping the membrane and using the mouse IgG anti-bradykinin (recognizes R_363_-R_371_ sequence in H-kininogen), a 115 kDa band was observed corresponding to the intact H-kininogen containing the bradykinin domain. However, the 62 kDa band detected by the polyclonal anti-H-kininogen antibody was not identified by the monoclonal anti-bradykinin antibody. In Fig. 1C in CHO-K1 cells, the rabbit IgG anti-H-kininogen indicates that in 0 min time point H-kininogen 115 kDa in size was detected (95.5 ± 1.5%, n = 5) and a fragment of 62 kDa in size was also detected (2.0 ± 0.7%, n = 5); after 1 h incubation there was partial H-kininogen hydrolysis and the amount of 115 kDa band decreased significantly (78.0 ± 4.8%, n = 5, *P* < 0.0001) and the 62 kDa fragment in size increased but no significantly (7.0 ± 0.7%, n = 5); at the end of incubation (3 h) the H-kininogen 115 kDa in size remained uncleaved but decreased significantly (73.9 ± 3.7%, n = 5, *P* < 0.0001) and the 62 kDa fragment in size increased significantly (15.2 ± 3.6%, n = 5, *P* < 0.0001). Similarly, Fig. 1D demonstrates that after stripping the membrane, the mouse IgG anti-bradykinin antibody detected the bradykinin domain inside H-kininogen (115 kDa), indicating the integrity of the samples. However, the 62 kDa band detected by the polyclonal anti-H-kininogen antibody was not identified by the monoclonal anti-bradykinin antibody.

Confluent CHO-K1 or CHO-745 cells in 35-mm plates were incubated with intact H-kininogen (200 nM) in a single volume of HEPES-Tyrode pH 7.35, without BSA, in the absence of kininases inhibitors. At the end of a specific time point (0, 10 min, 20 min, 30 min, 1 h, 2 h, 3 h) an aliquot of the incubation buffer was removed and mixed with SDS-PAGE sample buffer containing the reducing agent β-mercaptoethanol. Immunoblot studies were performed using rabbit IgG anti-H-kininogen, and after detection by chemiluminescence, the first antigen-antibody complex was removed. The same PVDF membrane was incubated with mouse IgG anti-bradykinin (recognizes the peptide in H-kininogen R363-R371), and the complex was detected by chemiluminescence. (A) and (B) represent CHO-745 cells developed with rabbit IgG anti-H-kininogen or mouse IgG anti-bradykinin, respectively; (C) and (D) represent CHO-K1 cells developed with rabbit IgG anti-H-kininogen or mouse IgG anti-bradykinin, respectively. The calculated molecular weights (kDa) are presented on the right side of all panels.

**Fig 2:**
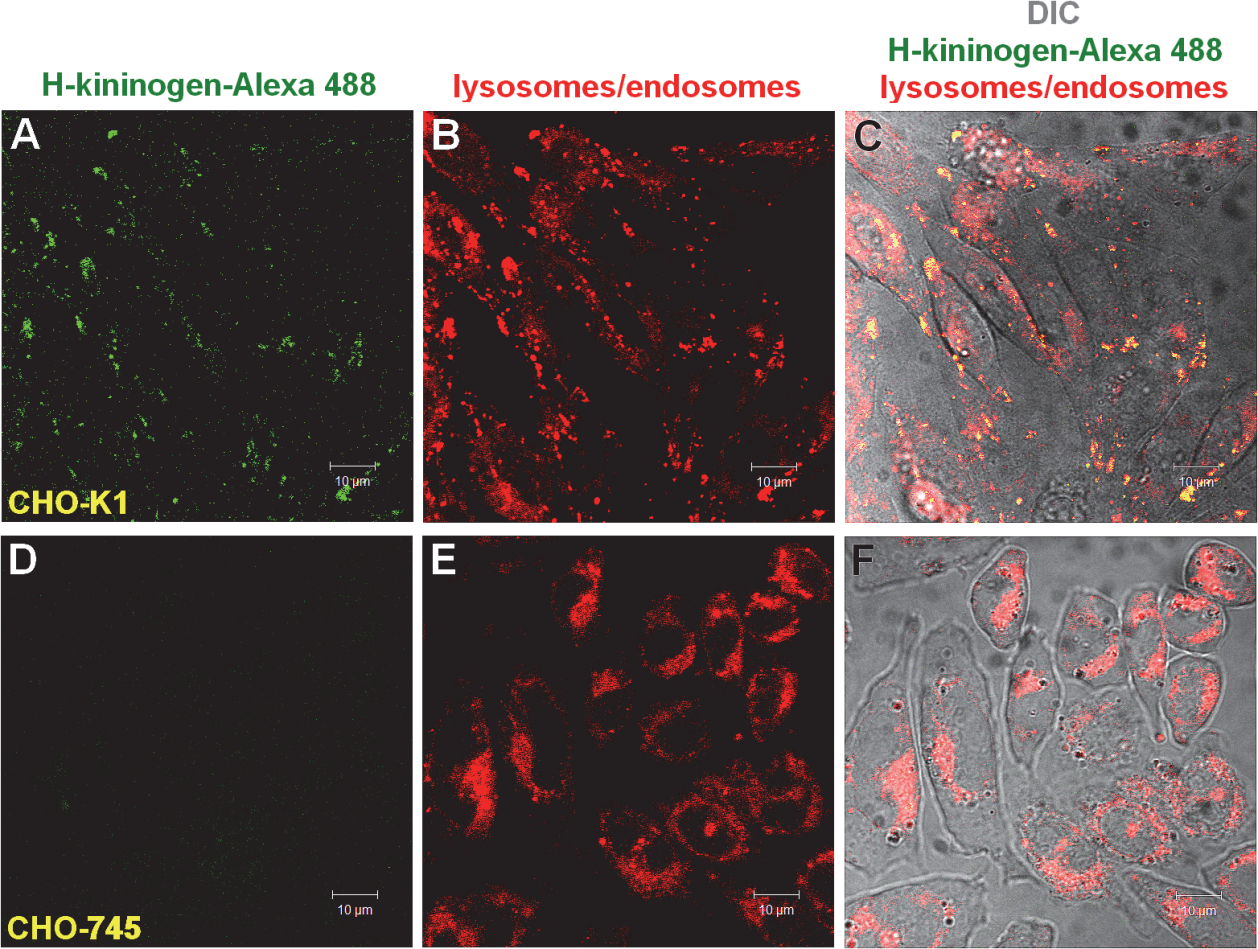
H-kininogen internalization in the presence of zinc and PGs.

Fig. 2 demonstrates that the H-kininogen-Alexa 488 endocytosis was strongly dependent on the presence of proteoglycans at the cell surface. In the wild type CHO-K1 cells, the H-kininogen-Alexa 488 endocytosis (2,719.00 ± 0.001 pixels/cells) was approximately 10-fold higher than the endocytosis in the CHO-745 mutant defective in proteoglycans biosynthesis (225.00 ± 0.001 pixels/cell).

Lysosomes/endosomes of living CHO-K1 or CHO-745 cells grown on coverslips were labeled with 0.5 μM LT Red DND-99 in serum-free F12 medium with 50 μM Zn^2+^ at 37°C for 20 min. After washing with serum-free F12 medium plus 50 μM Zn^2+^, the cells were incubated with H-kininogen-Alexa 488 (80 nM) diluted in serum-free F12 medium plus 50 μM Zn^2+^. The fluorescent signals of LT DND-99 (red) and Alexa 488 (green) as well as the phase contrast micrographs were monitored in real time at 37°C with a confocal laser scanning microscope every 5 min and the figures correspond to 30 min of endocytosis. Endocytosis by CHO-K1 cells in the presence of zinc: H-kininogen-Alexa 488 (A), lysosomes/endosomes labeled with LT Red DND-99 (B), merged images and diphasic contrast (C); absence of H-kininogen endocytosis by CHO-745 cells in the presence of zinc: H-kininogen-Alexa 488 (D), lysosomes/endosomes labeled with LT Red DND-99 (E), merged images and diphasic contrast (F). The orange-yellow signals are indicative of colocalization.

**Fig 3:**
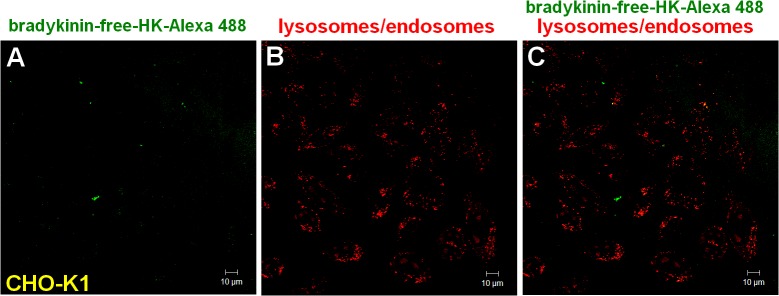
Endocytosis of bradykinin-free H-kininogen by CHO-K1 cells.

However, Fig. 3 demonstrates that the bradykinin-free H-kininogen-Alexa 488 (green) and LT Red DND-99 did not colocalize within endosomal acidic vesicles in CHO-K1 (45.5 ± 0.001 pixels/cell).

Lysosomes/endosomes of living CHO-K1 cells grown on coverslips were labeled with 0.5 μM LT Red DND-99 in serum-free F12 medium with 50 μM Zn^2+^ at 37°C for 20 min. After washing with serum-free F12 medium plus 50 μM Zn^2+^, the cells were incubated with bradykinin-free H-kininogen-Alexa 488 (80 nM) diluted in serum-free F12 medium plus 50 μM Zn^2+^. The fluorescent signals of LT DND-99 (red) and Alexa 488 (green) as well as the phase contrast micrographs were monitored in real time at 37°C with a confocal laser scanning microscope every 5 min and the figures correspond to 30 min of endocytosis. Normal endocytosis: (A) bradykinin-free H-kininogen-Alexa 488, (B) lysosomes/endosomes labeled with LT Red DND-99, (C) merged images and diphasic contrast. The orange-yellow signals are indicative of colocalization.

**Fig 4:**
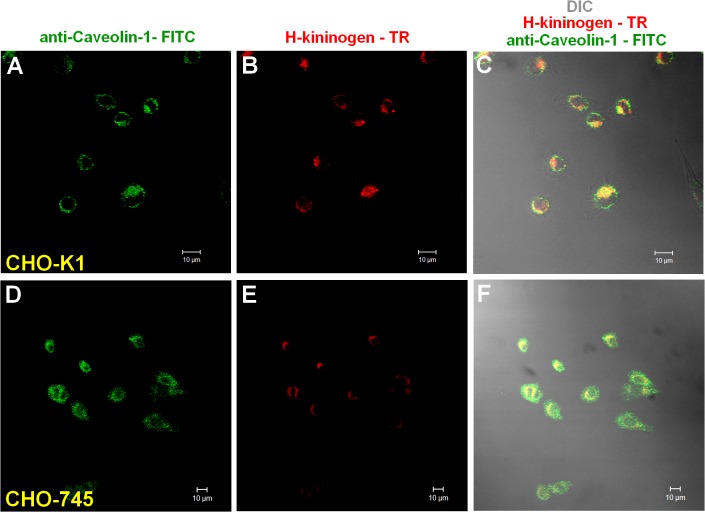
Cell surface colocalization of H-kininogen and caveolin.

The involvement of caveola in H-kininogen endocytosis by CHO-K1 was evaluated in serum-free F12 medium with 50 μM Zn^2 +^. Fig. 4 demonstrates that in CHO-K1 cells (A-C), caveolin was labeled by rabbit IgG anti-caveolin-1 (163.00 ± 0.002 pixels/cells) and was 57.5% colocalized with biotin-H-kininogen (93.70 ± 0.002 pixels/cell). In CHO-745 (D-F), caveolin was labeled (436.70 ± 0.002 pixels/cell), but the colocalization with biotin-H-kininogen was 41.1% (179.70 ± 0.002 pixels/cell).

CHO-K1 or CHO-745 cells grown on coverslips were incubated with biotin-H-kininogen (200 nM) diluted in serum-free F12 medium plus 50 μM Zn^2+^ for 1 h at 37°C. The cells were fixed and permeabilized, and rabbit IgG anti-caveolin-1 was added. Detection was achieved using a donkey IgG anti-rabbit IgG-FITC conjugated antibody. The biotin-H-kininogen detection was achieved with a streptavidin-Texas Red conjugated antibody. (A-C) Colocalization of H-kininogen and caveolin in CHO-K1 cells: (A) caveolin detected by rabbit IgG anti-caveolin-1 and donkey IgG anti-rabbit IgG-FITC conjugated antibodies (green); (B) biotin-H-kininogen developed with a streptavidin-Texas Red conjugated (red); and (C) composite of images presented in (A) and (B). (D-F) Colocalization of H-kininogen and caveolin in CHO-745 cells: (D) caveolin detected by rabbit IgG anti-caveolin-1 and donkey IgG anti-rabbit IgG-FITC conjugated antibodies (green); (E) biotin-H-kininogen developed with a streptavidin-Texas Red conjugated (red); and (F) composite of images presented in (D) and (E). The orange-yellow signals are indicative of colocalization.

**Fig 5:**
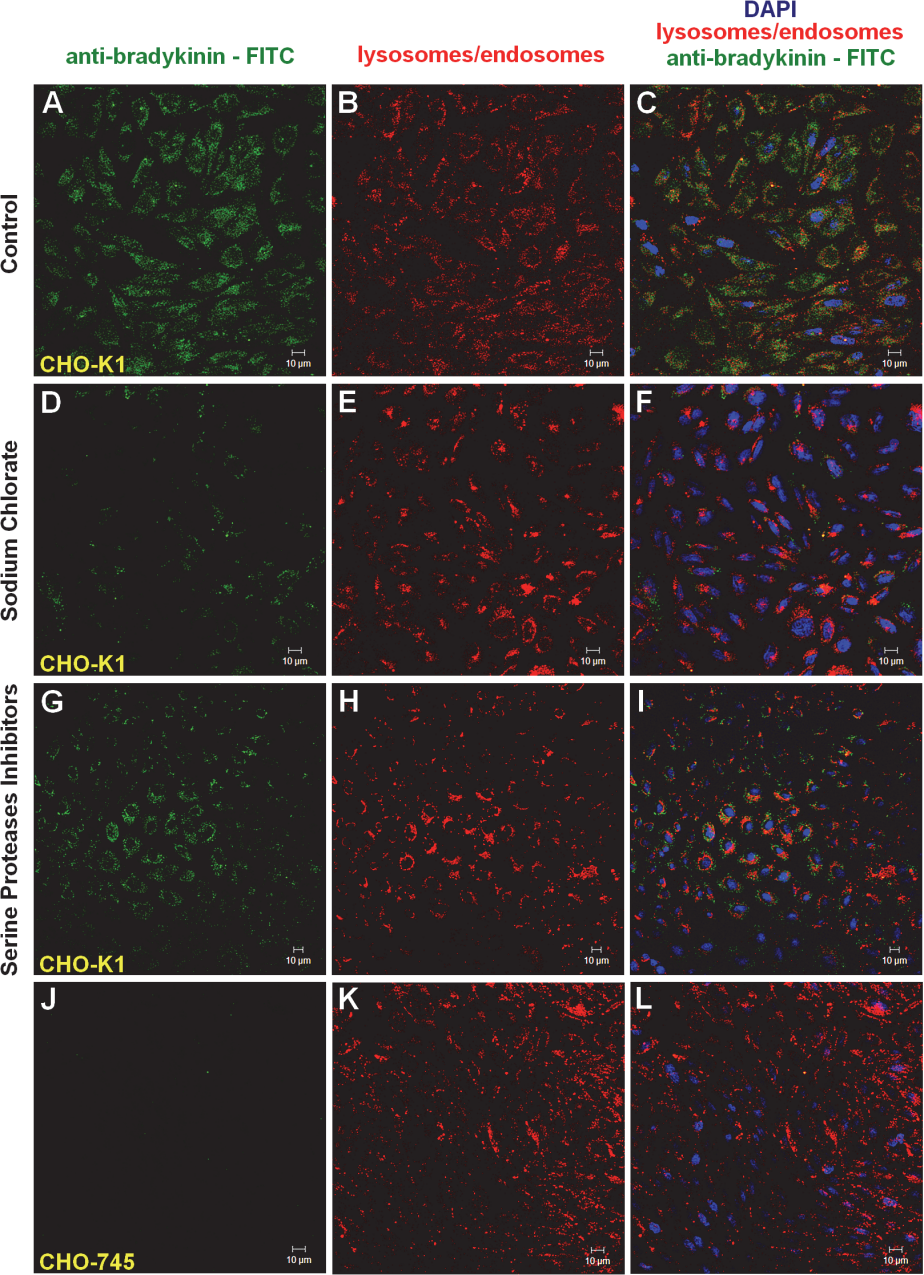
Endocytosis of H-kininogen containing the bradykinin domain by CHO-K1 cells.

The endocytosis of H-kininogen, containing the bradykinin domain, was evaluated by mouse IgG anti-bradykinin (recognizes R_363_-R_371_ sequence in H-kininogen). Fig. 5 depicts H-kininogen binding (344.0 ± 0.002 pixels/cell) and endocytosis (210.0 ± 0.001 pixels/cell) with colocalization with Lyso Tracker Red in acidic vesicles (61%) in CHO-K1 cells (Fig. 5 A-C). The effect of GAGs on the endocytosis process of H-kininogen containing the bradykinin domain was confirmed by the treatment of CHO-K1 cells with sodium chlorate (50 mM), an inhibitor of GAG sulfation [27]. Sodium chlorate treatment promoted a reduction in both H-kininogen binding (92.0 ± 0.002 pixels/cell) and endocytosis (64.0 ± 0.001 pixels/cell) in CHO-K1 cells (Fig. 5 D-F), with 70% colocalization in acidic vesicles with Lyso Tracker Red. In the presence of serine protease inhibitors (Fig. 5 G-I), CHO-K1 bound H-kininogen (351.0 ±0.002 pixels/cell), followed by endocytosis (160.0 ± 0.001 pixels/cell) and 45% colocalization with Lyso Tracker Red inside acidic vesicles. In CHO-745 cells (Fig. 5 J-L), lower H-kininogen binding (172.0 ± 0.002 pixels/cell) and endocytosis (97.0 ± 0.002 pixels/cell) were observed, with 56% colocalization in acidic vesicles with LysoTracker Red. These results demonstrate that the endocytosis of H-kininogen containing the bradykinin domain is mediated by GAG and the bradykinin release is not intracellular but occurs at the cell surface.

CHO-K1 or CHO-745 cells were grown on coverslips and incubated with LT Red DND-99 (0.5 μM) in serum-free F12 medium plus 50 μM Zn^2 +^ for 20 min to label lysosomes/endosomes. CHO-K1 cells were pretreated or not for 24 h with sodium chlorate (50 mM) diluted in medium plus 50 μM Zn^2+^. After LT Red DND-99 treatment, H-kininogen (200 nM) was added in the absence or presence of serine protease inhibitors (10 mM aprotinin, 1.0 mM PMSF and 100 mM benzamidine) diluted in serum-free F12 medium plus 50 μM Zn^2+^ for 3 h at 37°C. The cells were fixed and permeabilized, and bradykinin was detected by mouse IgG anti-bradykinin (recognizes the peptide in H-kininogen R363-R371) complexed to goat IgG anti-mouse FITC-conjugated antibody. The cells were examined by confocal fluorescence microscopy. The endocytosis and intracellular localization of H-kininogen containing the bradykinin domain (green) are indicated by colocalization with acidic vesicles previously labeled with LT Red DND-99 (red). Normal endocytosis by CHO-K1: H-kininogen containing bradykinin (A), lysosomes/endosomes labeled with LT Red DND-99 (B), and merged images (C); CHO-K1 cells treated with sodium chlorate: H-kininogen containing bradykinin (D), lysosomes/endosomes labeled with LT Red DND-99 (E), and merged images (F); CHO-K1 cells in presence of serine protease inhibitors: H-kininogen containing bradykinin (G), lysosomes/endosomes labeled with LT Red DND-99 (H), and merged images (I); and absence of H-kininogen containing bradykinin endocytosis by CHO-745 cells: H-kininogen containing bradykinin (J), lysosomes/endosomes labeled with LT Red DND-99 (K), and merged images (L). The orange-yellow signals are indicative of colocalization and the nucleus was stained in blue with DAPI.

**Fig 6:**
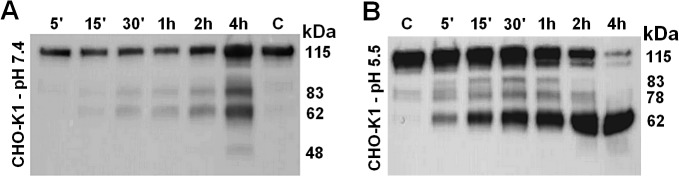
Kininogenase activity present in lysate fractions from CHO-K1 cells.

The lysate fraction of CHO-K1 cells was prepared after sonication as described in the Material and Methods section. Fig. 6 presents the kininogenase activity assayed at pH 7.4 or pH 5.5 over time. H-kininogen fragments were detected by rabbit IgG anti-H-kininogen. At pH 7.4 (Fig. 6A), H-kininogen (115 kDa) was partially hydrolyzed after 5 min incubation with the lysate fraction, and protein bands of 115 kDa (95.4%), 83 kDa (3.4%) and 62 kDa (1.3%) were detected. After 4 h, the protein bands detected were 115 kDa (44.2%), 83 kDa (23.6%), 62 kDa (28.7%) and 48 kDa (3.5%). At pH 5.5 (Fig. 6B), H-kininogen (115 kDa) was also partially hydrolyzed after 5 min incubation with the lysate fraction, yielding protein bands of 115 kDa (70.0%), 83 kDa (5.5%), 78 kDa (11.9%) and 62 kDa (12.7%). However, after 4 h, H-kininogen was hydrolyzed remaining 14.9% as 115 kDa and generating a fragment of 62 kDa in size (73.0%).

A single volume of sample was prepared, maintaining the proportion of intact H-kininogen (1.0 mg) to CHO-K1 lysate fraction (3.5 mg). All samples were incubated in buffer at pH 7.4 or pH 5.5 at 37°C. At predetermined times (5 min, 15 min, 30 min, 1 h, 2 h, 4 h), an aliquot of incubation sample was withdrawn, and the reactions were stopped with sample buffer for SDS-PAGE under reducing conditions and frozen immediately. After the protein was electroblotted onto PVDF membranes, a primary antibody and a secondary antibody were used. The antigen-antibody complex was developed with the SuperSignal chemiluminescent substrate. (A) HEPES 20.0 mM, NaCl 5.0 mM, pH 7.4 and (B) sodium acetate 50.0 mM, pH 5.5. Control sample “C”: H-kininogen (1.0 μg) 4 h incubation in the buffer. The calculated molecular weights (kDa) are presented on the right side.

**Fig 7:**
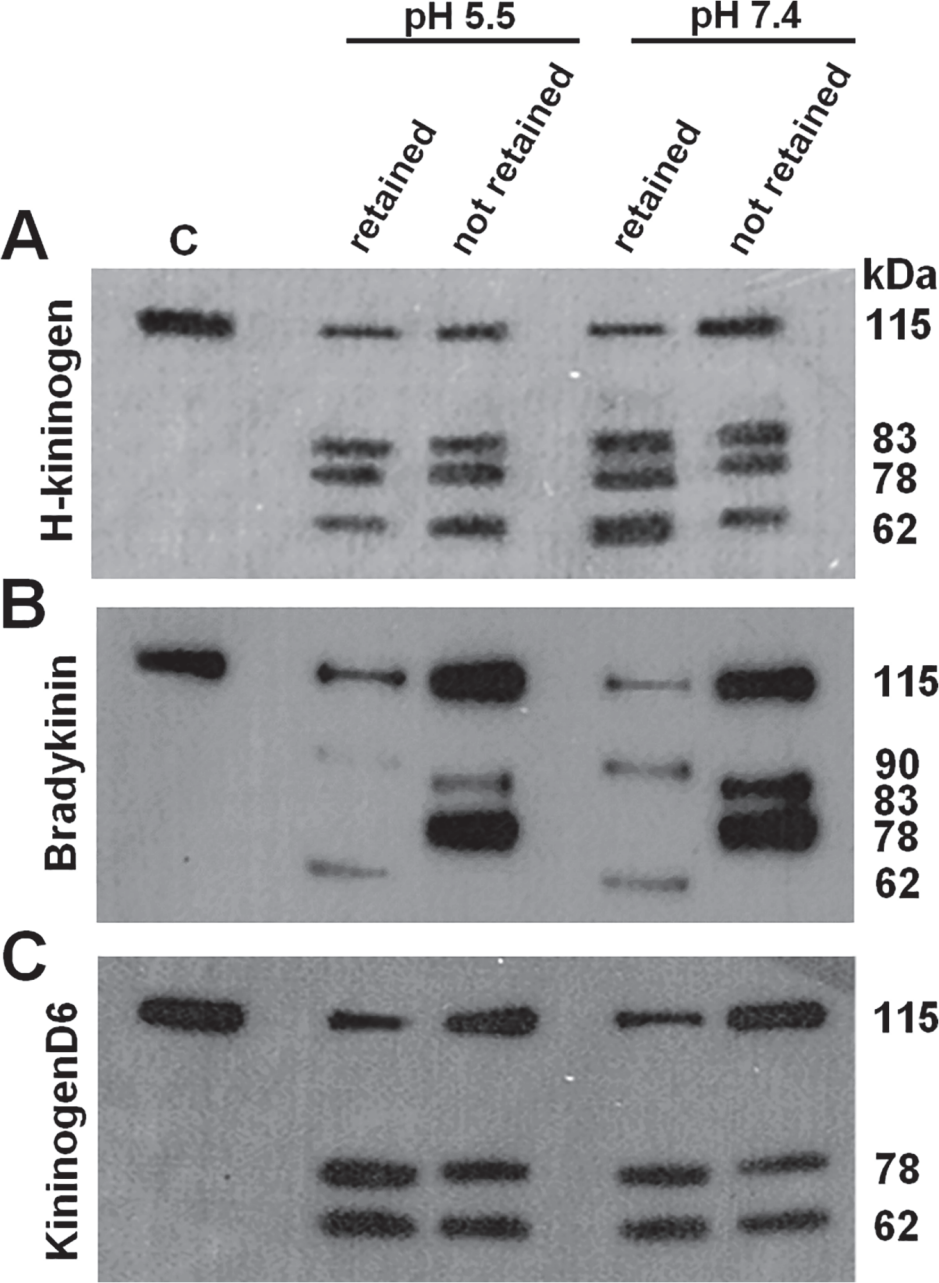
Immunoblotting of H-kininogen cleavage by lysate fractions from CHO-K1 cells separated by affinity chromatography.

In an attempt to separate the enzymes present in the lysate fraction prepared from CHO-K1, antipain was used as a ligand in affinity chromatography. The fractions retained or not retained on the resin were tested for their kininogenase activity. The analysis of H-kininogen or its fragments was accomplished by switching antibodies after stripping the same membrane. The experiment was performed in triplicate. To confirm the results, the order of addition and removal of antibodies was switched. The antibodies used included rabbit IgG anti-H-kininogen, mouse IgG anti-bradykinin and mouse IgG anti-kininogenD6 (recognizes S_543_-M_554_ sequence in H-kininogen domain6). Fig. 7 presents the experiments performed with the lysate fractions of CHO-K1 retained or not by antipain-Sepharose assayed at pH 5.5 or pH 7.4. Fig. 7A demonstrates that the rabbit IgG anti-H-kininogen detected protein bands of 115 kDa, 83 kDa, 78 kDa and 62 kDa, indicating that H-kininogen was hydrolyzed partially, which was similar at both pHs, for both lysate fractions, and for samples retained or not on the resin. Fig. 7B demonstrates that the mouse IgG anti-bradykinin detected, at both pHs, that the fraction not retained partially hydrolyzed H-kininogen, with protein bands of 115 kDa, 83 kDa and 78 kDa, indicating bradykinin. The fraction retained on the resin hydrolyzed H-kininogen at both pHs, releasing bradykinin more efficiently. Finally, Fig. 7C demonstrates that mouse IgG anti-kininogenD6 detected the 78 kDa and 62 kDa fragments at both pHs and in both lysate fractions, retained or not on the resin, indicating that the protein bands contain the amino acid sequence corresponding to S_543_-M_554_ (D6 domain). The pH of the buffer did not influence the activity of either lysate fraction, retained and not retained on antipain-Sepharose, and both lysate fractions differed from each other with respect to bradykinin release.

H-kininogen (1.0 μg) was incubated at pH 5.5 or pH 7.4 with lysate fractions from CHO-K1 cells (4.0 μg), retained or not on antipain-Sepharose, for 4 h at 37°C. The proteins under reducing conditions were separated by 13% SDS-PAGE. A primary antibody and secondary antibody were used, and the antigen-antibody complex was developed with the SuperSignal chemiluminescent substrate. In an attempt to characterize the fragments of H-kininogen, different primary antibodies were used after the first development by stripping the antigen-antibody complex from the PVDF membrane according to the Methods section. (A) the first antibody is rabbit IgG anti-H-kininogen; (B) the first antibody is mouse IgG anti-bradykinin (recognizes the peptide in H-kininogen R363-R371); and (C) the first antibody is mouse IgG anti-kininogenD6 (recognizes S_543_-M_554_ sequence in H-kininogen domain6). Control sample “C”: H-kininogen (1. 0 μg). The calculated molecular weights (kDa) are presented on the right side.

## Discussion

The present work demonstrates that the bradykinin domain present in H-kininogen promotes endocytosis mediated by proteoglycans and there are different kininogenases inside CHO cells. Considering the kinin release in CHO-K1 incubation buffer that has been previously reported [17] and comparing these findings to the kinin release in CHO-745 incubation buffer in the present report, our data clearly demonstrate that at pH 7.35, serine or cysteine proteases on the cell surface hydrolyze H-kininogen, thereby releasing bradykinin (Fig. 1). The bradykinin release by cysteine proteases may be controlled by GAGs/PGs, consistent with the regulation of these enzymes by GAGs as previously reported [28]. Moreover, H-kininogen binding to heparan and chondroitin sulfate GAGs efficiently interferes with bradykinin release by kallikrein, a serine protease, in plasma and on endothelial surfaces [29]. In endothelial cells, bradykinin stimulates the synthesis of HSPG secreted into the medium and produces an anti-mitogenic effect on the cell cycle [30].

In CHO-K1 cells, the internalization of the H-kininogen containing the bradykinin domain is mediated by GAGs/PGs (Fig. 2). The inhibition of GAG sulfation blocks endocytosis, and in the presence of serine protease inhibitors, no significant change in endocytosis is detected. Thus, intact H-kininogen internalization is dependent on GAGs (Fig. 5).

Tumor cells can modulate tumor progression by processing H-kininogen and releasing proangiogenic bradykinin and anti-angiogenic bradykinin-free H-kininogen [7]. According to our data, the process can occur on lipid raft domains/caveolae, as demonstrated by the colocalization of H-kininogen with caveolin-1 (Fig. 4). In our previous work we demonstrated that in CHO-K1 pretreated with methyl-β-cyclodextrin H-kininogen endocytosis is prevented and it did not colocalize with transferrin in the early phase of the endocytic process strongly indicating that the endocytosis is not dependent on clathrin [17]. Consistent with our findings, FXII binding to HUVECs depends on intact caveolae on the cellular surface [31], and caveolae harbor proteoglycans [32]. The bradykinin-free H-kininogen does not internalize as shown in the present work (Fig. 3) and as recently reported by our group [33], and it can act as anti-angiogenic molecule, as previously reported [34].

The plasma and tissue kallikrein-kinin systems represent a metabolic cascade that, when activated, triggers the release of vasoactive kinins [35]. The kallikreins are serine proteases divided into the two major categories of plasma kallikrein and kallikrein-related peptidases, which differ significantly in their location in the plasma or tissues, respectively, and in their molecular weights, substrate specificity, gene structure and kinin generated [36], [37]. Some proteases from the cysteine protease family have also been described as kininogenases, but not all of them release kinins [38]. Using purified proteins *in vitro*, our group demonstrated H-kininogen hydrolysis by cathepsin B at three sites, generating fragments of 80 kDa, 60 kDa and 40 kDa at pH 6.3 [39]. Moreover, in human colon cancer cells, caveolin-1 affects the expression and localization of cathepsin B and pro-urokinase and their receptors, thereby mediating cell-surface proteolytic events associated with invasion [40]. Furthermore, caveolin-1 enhances tissue kallikrein 6 secretion by these cells [41].

In our previous work, we reported cell-associated H-kininogen hydrolysis (fragment of 82 kDa) in CHO-K1 after 3 h incubation [17]. In the present work, we report that lysate fraction of CHO-K1 is more effective in the total cleavage of intact H-kininogen (115 kDa) at pH 5.5 after 4 h incubation. Nevertheless, the lysate fraction also possesses kininogenase activity at pH 7.4, producing fragments of 83 kDa, 62 kDa and 48 kDa, including intact H-kininogen at 115 kDa (Fig. 6). Because the hydrolysis patterns are so similar at pH 5.5 and pH 7.4, it is possible that a single kininogenase present in the crude lysates works with a pH optimum of 5.5 that can still work at pH 7.4, but more slowly.

These findings suggest the role of cysteine proteases in H-kininogen processing on the cell surface and in acidic endosomal vesicles. The affinity chromatography using antipain-Sepharose of the CHO-K1 lysate fraction was performed and successfully separated kininogenases. There was no difference in the assays at pH 5.5 and 7.4, but the proteins in the fraction bound to resin released bradykinin from H-kininogen, whereas the proteins in the fraction unbound to resin cleaved H-kininogen at other sites but did not release bradykinin (Fig. 7).

Taken together, the data in the present work support the hypothesis that after blood vessel injury, H-kininogen can interact and bind to the surface of non-endothelial cells. On lipid raft domains/caveolae or endosomes, PGs mediate the endocytosis of intact H-kininogen, which can be processed by different classes of proteases [42] [43]. The cleavage produces H-kininogen fragments that may play roles as cystatins, effectors of the innate immunity, angiogenesis and coagulation [44].

## References

[1] Maurer M, Bader M, Bas M, Bossi F, Cicardi M, Cugno M, et al New topics in bradykinin research. Allergy 2011;66: 1397–1406. 10.1111/j.1398-9995.2011.02686.x 21859431

[2] Rocha e, Silva M, Beraldo WT, Rosenfeld G. Bradykinin, a hypotensive and smooth muscle stimulating factor released from plasma globulin by snake venoms and by trypsin. Am J Physiol 1949;156: 261–273. 18127230 10.1152/ajplegacy.1949.156.2.261

[3] Clapp C, Thebault S, Jeziorski MC, Martínez de la Escalera G. Peptide Hormone Regulation of Angiogenesis. Physiol Rev 2009;89: 1177–1215. 10.1152/physrev.00024.2009 19789380

[4] Cagliani R, Forni D, Riva S, Pozzoli U, Colleoni M, Bresolin N, et al Evolutionary analysis of the contact system indicates that kininogen evolved adaptively in mammals and in human populations. Mol Biol Evol 2013;30: 1397–408. 10.1093/molbev/mst054 23505046

[5] Kashuba E, Bailey J, Allsup D, Cawkwell L. The kinin-kallikrein system: physiological roles, pathophysiology and its relationship to cancer biomarkers. Biomarkers 2013;18: 279–296. 10.3109/1354750X.2013.787544 23672534

[6] Leeb-Lundberg FLM, Marceau F, Müller-Esterl W, Pettibone DJ, Zuraw BL. International Union of Pharmacology. XLV. Classification of the Kinin Receptor Family: from Molecular Mechanisms to Pathophysiological Consequences. Pharmacol Rev 2005;57: 27–77. 15734727 10.1124/pr.57.1.2

[7] Guo YL, Colman RW. Two faces of high-molecular-weight-kininogen (HK) in angiogenesis: bradykinin turns it on and cleaved HK (HKa) turns it off. J Thromb Haemost 2005;3: 670–676. 15733059 10.1111/j.1538-7836.2005.01218.x

[8] McCrae KR, Doñate F, Merkulov S, Sun D, Qi X, Shaw DE. Inhibition of angiogenesis by cleaved high molecular weight kininogen (HKa) and HKa Domain 5. Curr Cancer Drug Targets 2005;5: 519–528. 16305348 10.2174/156800905774574039

[9] Bryant JW, Shariat-Madar Z. Human Plasma Kallikrein-Kinin System: Physiological and Biochemical Parameters. Cardiovas Hematol Agents Med Chem 2009;7: 234–250. 19689262 10.2174/187152509789105444 PMC4905712

[10] Kaplan AP, Joseph K, Shibayama Y, Nakazawa Y, Ghebrehiwet B, Reddigari S, et al Bradykinin formation. Plasma and tissue pathways and cellular interactions. Clin Rev Allergy Immunol 1998;16: 403–429. 9926288 10.1007/BF02737659

[11] Schmaier AH, McCrae KR. The plasma kallikrein–kinin system: its evolution from contact activation. J Thromb Haemost 2007;5: 2323–2329. 17883591 10.1111/j.1538-7836.2007.02770.x

[12] Kolte D, Osman N, Yang J, Shariat-Madar Z. High molecular weight kininogen activates B2 receptor signaling pathway in human vascular endothelial cells. J Biol Chem 2011;286: 24561–24571. 10.1074/jbc.M110.211557 21586566 PMC3137031

[13] Renné T, Dedio J, David G, Muller-Esterl W. High molecular weight kininogen utilizes heparan sulfate proteoglycans for accumulation on endothelial cells. J Biol Chem 2000;275: 688–696. 10.1074/jbc.M000313200 10843988

[14] Renné T, Muller-Esterl W. Cell surface-associated chondroitin sulfate proteoglycans bind contact phase factor H-kininogen. FEBS Lett 2001;500: 36–40. 11434922 10.1016/s0014-5793(01)02570-4

[15] Götte M. Syndecans in Inflammation. FASEB J 2003;17: 575–591. 12665470 10.1096/fj.02-0739rev

[16] Gozzo AJ, Motta G, Cruz-Silva I, Nunes VA, Barros NM, Carmona AK, et al Heparin affects the interaction of kininogen on endothelial cells. Biochimie 2011;93: 1839–1845. 10.1016/j.biochi.2011.07.003 21784122

[17] Melo KRB, Gutierrez A, Nascimento FD, Araújo MK, Sampaio MU, Carmona AK, et al Involvement of heparan sulfate proteoglycans in cellular uptake of high molecular weight kininogen. Biol Chem 2009;390: 145–155. 10.1515/BC.2009.016 19040351

[18] Esko JD, Stewart TE, Taylor WH. Animal cell mutants defective in glycosaminoglycan biosynthesis. Proc Natl Acad Sci USA 1985;82: 3197–3201. 3858816 10.1073/pnas.82.10.3197 PMC397742

[19] Vogel R, Kaufmann J, Chung DW, Kellermann J, Müller-Esterl W. Mapping of the prekallikrein-binding site of human H-kininogen by ligand screening of lambda gt11 expression libraries. Mimicking of the predicted binding site by anti-idiotypic antibodies. J Biol Chem 1990;265: 12494–502. 1695630

[20] Motta G, Sampaio CAM, Sampaio UM. Human plasma kallikrein. Immunoreactivity and activity on natural and synthetic substrates. Agents Actions Suppl 1992;36: 200–208. 1609643

[21] Shimamoto K, Ando T, Nakao T, Tanaka S, Sakuma M, Miyahara M. A sensitive radioimmunoassay method for urinary kinins in man. J Lab Clin Med 1978;91: 721–728. 641396

[22] Motta G, Shariat-Madar Z, Mahdi F, Sampaio CAM, Schmaier AH. Assembly of high molecular weight kininogen and activation of prekallikrein on cell matrix. Thromb Haemost 2001;86: 840–847. 11583317

[23] Michaud D. Gel electrophoresis of proteolytic enzymes. Anal Chim Acta 1998;372: 173–185.

[24] Toyo-Oka T, Masaki T. Calcium-activated neutral protease from bovine ventricular muscle: isolation and some of its properties. J Mol Cell Cardiol 1979;11: 769–786. 40039 10.1016/0022-2828(79)90402-4

[25] Laemmli UK. Cleavage of structural proteins during the assembly of the head of bacteriophage T4. Nature 1970;227: 680–685. 5432063 10.1038/227680a0

[26] Durrant I, Fowler S. Chemiluminescent detection systems for protein blotting In: Dunbar B.S. editor. Protein blotting: a practical approach. Oxford University Press Inc., New York; 1996 pp. 141–151.

[27] Hoogewerf AJ, Cisar LA, Evans DC, Bensadoun A. Effect of chlorate on the sulfation of lipoprotein lipase and heparan sulfate proteoglycans. Sulfation of heparan sulfate proteoglycans affects lipoprotein lipase degradation. J Biol Chem 1991;266: 16564–16571. 1885587

[28] Almeida PC, Nantes IL, Chagas JR, Rizzi CCA, Faljoni-Alario A, Carmona E, et al Cathepsin B activity regulation. Heparin-like glycosaminoglycans protect human cathepsin B from alkaline pH-induced inactivation. J Biol Chem 2001;276: 944–951. 11016923 10.1074/jbc.M003820200

[29] Renné T, Schuh K, Müller-Esterl W. Local bradykinin formation is controlled by glycosaminoglycans. J Immunol 2005;175: 3377–3385. 16116231 10.4049/jimmunol.175.5.3377

[30] Moreira CR, Porcionatto MA, Dietrich CP, Nader HB. Effect of bradykinin and PMA on the synthesis of proteoglycans during the cell cycle of endothelial cells in culture. Int Immunopharmacol 2003;3: 293–298. 12639806 10.1016/S1567-5769(02)00262-X

[31] Schousboe I, Thomsem P, van Deurs B. Factor XII binding to endothelial cells depends on caveolae. Eur J Biochem 2004;271: 2998–3005. 15233796 10.1111/j.1432-1033.2004.04229.x

[32] Donatello S, Babina IS, Hazelwood LD, Hill AD, Nabi IR, Hopkins AM. Lipid raft association restricts CD44-ezrin interaction and promotion of breast cancer cell migration. Am J Pathol 2012;181: 2172–2187. 10.1016/j.ajpath.2012.08.025 23031255 PMC3502863

[33] Veronez CL, Nascimento FD, Melo KRB, Nader HB, Tersariol ILS, Motta G. The involvement of proteoglycans in the human plasma prekallikrein interaction with the cell surface. PLoS One 2014 March 9(3):e91280 10.1371/journal.pone.0091280 24621563 PMC3951348

[34] Colman RW. Regulation of angiogenesis by the kallikrein-kinin system. Curr Pharm Des 2006;12: 2599–2607. 16842160 10.2174/138161206777698710

[35] Moreau ME, Garbacki N, Molinaro G, Brown NJ, Marceau F, Adam A. The kallikrein-kinin system: current and future pharmacological targets. J Pharmacol Sci 2005;99: 6–38. 16177542 10.1254/jphs.srj05001x

[36] Pathak M, Wong SS, Dreveny I, Emsley J. Structure of plasma and tissue kallikreins. Thromb Haemost 2013;110: 423–433. 10.1160/TH12-11-0840 23494059

[37] Goettig P, Magdolen V, Brandstetter H. Natural and synthetic inhibitors of kallikrein-related peptidases (KLKs). Biochimie 2010;92: 1546–1567. 10.1016/j.biochi.2010.06.022 20615447 PMC3014083

[38] Veillard F, Lecaille F, Lalmanach G. Lung cysteine cathepsins: intruders or unorthodox contributors to the kallikrein-kinin system? Int J Biochem Cell Biol 2008;40: 1079–1094. 18093865 10.1016/j.biocel.2007.10.030

[39] Barros NMT, Tersariol ILS, Oliva MLV, Araújo MS, Sampaio CAM, Juliano L, et al High molecular weight kininogen as substrate for cathepsin B. Biol Chem 2004;385: 551–555. 15255189 10.1515/BC.2004.066

[40] Cavallo-Medved D, Mai J, Dosescu J, Sameni M, Sloane BF. Caveolin-1 mediates the expression and localization of cathepsin B, pro-urokinase plasminogen activator and their cell-surface receptors in human colorectal carcinoma cells. J Cell Sci 2005;118: 1493–1503. 15769846 10.1242/jcs.02278

[41] Henkhaus RS, Roy UKB, Cavallo-Medved C, Sloane BF, Gerner EW, Ignatenko NA. Caveolin-1-mediated expression and secretion of kallikrein 6 in colon cancer cells. Neoplasia 2008;10: 140–148. 18283336 10.1593/neo.07817 PMC2244689

[42] Cudic M, Fields GB. Extracellular proteases as targets for drug development. Curr Protein Pept Sci 2009;10: 297–307. 19689354 10.2174/138920309788922207 PMC4339022

[43] Roycik MD, Fang X, Sang Q-X. A fresh prospect of extracellular matrix hydrolytic enzymes and their substrates. Curr Pharm Des 2009;15: 1295–1308. 19355969 10.2174/138161209787846676

[44] Lalmanach G, Naudin C, Lecaille F, Fritz H. Kininogens: More than cysteine protease inhibitors and kinin precursors. Biochimie 2010;92: 1568–1579. 10.1016/j.biochi.2010.03.011 20346387

